# Supplementary material for: Chemical Characterization of Plant Extracts and Evaluation of their Nematicidal and Phytotoxic Potential
Source: Molecules. 2021 Apr 12;26(8):2216. doi: 10.3390/molecules26082216 (PMC8070253; doi:10.3390/molecules26082216)
Supplement: Supplementary file 1 [file molecules-26-02216-s001.zip › molecules-1147499-supplementary/Supplementary material.docx]

**SUPPLEMENTARY MATERIAL**

**Chemical characterization of plant extracts and evaluation of their nematicidal and phytotoxic potential**

Raúl Velasco-Azorsa,^1^ Héctor Santiago-Cruz,^2^, Ignacio Cid del Prado-Vera,^3^ MarcoVinicio Ramirez-Mares,^4^ María del Rocío Gutiérrez-Ortiz,^5^ Norma Francenia Santos-Sánchez, ^2^ Raúl Salas-Coronado,^2^ Claudia Villanueva-Cañongo,^2^ Karla Isabel Lira de León,^6^ and Beatriz Hernández-Carlos^2,^*

^1^ Instituto de Recursos, Universidad del Mar, Puerto Ángel, San Pedro Pochutla, Oaxaca 70902, México; rvazorsa@gmail.com

^2^ Instituto de Agroindustrias, Universidad Tecnológica de la Mixteca, Acatlima, Huajuapan de León, Oaxaca 69000, México; [sacrumix@hotmail.com](mailto:sacrumix@hotmail.com), [nsantos@mixteco.utm.mx](mailto:nsantos@mixteco.utm.mx), [rsalas@mixteco.utm.mx](mailto:rsalas@mixteco.utm.mx), [villanueva.vc@gmail.com](mailto:villanueva.vc@gmail.com), [bhcarlos@mixteco.utm.mx](mailto:bhcarlos@mixteco.utm.mx)

^3^ Colegio de Postgraduados, km 36.5 Carretera México-Texcoco, Montecillos. Estado de México, CP 56230, México; [icid@colpos.mx](mailto:icid@colpos.mx)

^4^ Departamento de Ingeniería Química y Bioquímica, Tecnológico Nacional de México/I.T. Morelia, Av. Tecnológico 1500, Lomas de Santiaguito, Morelia 58120, Michoacán, México; [jvinicio2000@yahoo.com.mx](mailto:jvinicio2000@yahoo.com.mx)

^5^ Instituto de Ecologia, Universidad del Mar, Puerto Ángel, San Pedro Pochutla, Oaxaca 70902, México; [rocio@angel.umar.mx](mailto:rocio@angel.umar.mx)

^6^  Facultad de Química, Universidad Autónoma de Querétaro, Las Campanas, Queretaro 76010, Mexico; [liraleonki@gmail.com](mailto:liraleonki@gmail.com)

* Correspondence: [bhcarlos@mixteco.utm.mx](mailto:bhcarlos@mixteco.utm.mx)

**Abstract**: *Nacobbus aberrans* ranks among the “top ten” plant-parasitic nematodes of phytosani-tary importance. It causes significant losses in commercial interest crops in America and is a potential risk in the European Union. The nematicidal and phytotoxic activities of seven plant extracts against *N. aberrans* and *Solanum lycopersicum* were evaluated in vitro, respectively. The chemical nature of three nematicidal extracts (EC_50,48h_ ≤ 113 µgmL^-1^) was studied through NMR analysis. Plant extracts showed nematicide activity on second-stage juveniles (J2): (≥87%) at 1,000 µgmL^-1^ after 72 h, and their EC_50_ values were 71.4 - 468.1 and 31.5 - 299.8 µgmL^-1^ after 24 and 48 h, respectively. Extracts with the best nematicide potential (EC_50,48h_ < 113 µgmL^-1^) were those from *Adenophyllum aurantium, Alloispermum integrifolium, and Tournefortia densiflora*, which inhibited *L. esculentum* seed growth by 100 % at 20 µgmL^-1^. Stigmasterol (**1**), β-sitosterol (**2**), α-terthienyl (**3**) were identified from *A. aurantium*, while **1**, **2**, lutein (**4**), centaurin (**5**), patuletin-7-β-*O*-glucoside (**6**), pendulin (**7**), and penduletin (**8**) were identified from *A. integrifolium*. From *T. densiflora* extract, allantoin (**9**), 9-O-angeloyl-retronecine (**10**), and its N-oxide (**11**) were identified. The present research is the first to report the effect of *T. densiflora, A. integrifolium,* and *A. aurantium* against *N. aberrans* and *S*. *lycopersicum* and chemically characterized nematicide extracts that may provide alternative sources of botanical nematicides.

**Table S1**. Effect of plant extracts at 10 ppm on immobility of *N. aberrans* J2s after different exposure times

| Treatment | % Immobility J2s | | | | | | | | |  |  |
| --- | --- | --- | --- | --- | --- | --- | --- | --- | --- | --- | --- |
|  | 12h | 24h | 36h | | 48h | | 60h | 72h | | | |
| *A. aurantium* A | 1.3±13 a | 4.9±11 ab | | 7.7±11 ab | 19.7±9 ab | 20.2±13 ab | | | 25.9±16 b | |  |
| *A. aurantium* R | 16.8±13 a | 11.6±12 a | | 14.5± 10 a | 26.8±9 a | 17.2±11 a | | | 26.9±10 a | |  |
| *A. cuspidata* | -7.0±10 ab | -15.6±13 a | | 5.5±6 b | 8.9±8 b | 3.6±9 b | | | 7.13±7 b | |  |
| *A. integrifolium* | 9.3±10 a | 7.5±8 a | | 8.6±8 a | 22.7±6 a | 21.0±12 a | | | 22.0±7 a | |  |
| *A. subviscida* | -12.9±19 a | -14.2±18 a | | -1.6±13 ab | 4.3±15 b | 2.8±14 b | | | 0.7±21 b | |  |
| *G. mexicanum* | 5.0±11 a | 6.5±11 a | | 7.1±13 a | 11.7±11 a | 14.4±12 a | | | 5.4±9 a | |  |
| *H. terebinthinaceous* | 4.9±8 a | 3.4±8 a | | 4.2±6 a | 11.9± a | 4.0±14 a | | | 10.0±8 a | |  |
| *T. densiflora* R | -1.9±0 ac | -22.8±15 a | | -15.2±12 ab | 11.1±5 b | 8.5±6 b | | | 6.9±6 b | |  |
| *T. densiflora* A | -0.9±1 a | -0.1±4 a | | 0.4±3 a | 7.71±6 a | 6.3±5 a | | | 6.5±6 a | |  |

* Immobility recovery

A: Stem part, R: roots

In each row, data followed by the same letter are not significantly different according to Tukey’s test (p<0.05); mean of all values

**Table S2**. ^1^H (400 MHz) and ^13^C (100 MHz) data for compounds **5**-**8**. CD_3_OD

|  | **5** | | **6** | | **7** | | **8** | |
| --- | --- | --- | --- | --- | --- | --- | --- | --- |
| Atom | δ ^1^H | δ^13^C | δ 1H | ^13^C | δ ^1^H | ^13^C | δ ^1^H | ^13^C |
| 1 |  |  |  |  |  |  |  |  |
| 2 |  | 158.2 |  | 154.4 |  | 154.1 |  | 157.8 |
| 3 |  | 139.8 |  | 146.6 |  | 140.0 |  | 138.6 |
| 4 |  | 180.3 |  | 178.2 |  | 180.4 |  | 179.0 |
| 5 |  | 153.7 |  | 153.4 |  | 153.8 |  | 156.3 |
| 6 |  | 133.7 |  | 133.6 |  | 133.4 |  | 132.1 |
| 7 |  | 157.9 |  | 160.0 |  | 160.7 |  | 159.9 |
| 8 | 6.83s | 95.6 | 6.90 s | 95.7 | 6.75 s | 92.2 | 6.82 s | 90.7 |
| 9 |  | 153.7 |  | 153.4 |  | 154.1 |  | 152.7 |
| 10 |  | 108.1 |  | 107.0 |  | 107.4 |  | 106.0 |
| 1' |  | 123.9 |  | 124.2 |  | 124.1 |  | 124.0 |
| 2' | 7.58 sa | 112.2 | 6.90 sa | 116.5 | 8.09 d (8.6) | 131.3 | 8.13 d (8.9) | 129.9 |
| 3' | - | 147.6 |  | 146.2 | 7.24 d (8.6) | 117.7 | 7.28 d (8.9) | 116.2 |
| 4' | - | 151.8 | _ | 149.3 |  | 161.3 |  | 159.3 |
| 5' | 6.99 d (8.7) | 116.2 | 7.77 d (2.2) | 116.5 | 7.24 d (8.6) | 117.7 | 7.28 d (8.9) | 116.2 |
| 6' | 7.60 d (8.7) | 122.4 | 7.68 dd (8.3, 2.2) | 122.2 | 8.09 d (8.6) | 131.3 | 8.13 d (8.9) | 129.9 |
| OCH3-3 | 3.79 | 60.5 | _ | _ | 3.80 | 60.6 | 3.84 | 59.7 |
| OCH3-6 | 3.88 | 61.5 | 3.88 | 61.8 | 3.83 | 60.7 | 3.86 | 59.3 |
| OCH3-7 | _ | _ | _ | _ | 3.96 | 57.1 | 3.99 | 55.6 |
| OCH3-4' | 3.91 | 56.4 | _ | _ | _ | _ | _ | _ |
| 7-Glc-1 | 5.11 d (7.2) | 101.9 | 5.11 d (7.4) | 102.3 |  |  |  |  |
| 4' -Glc-1 | _ | _ | _ | _ |  | 101.8 | _ | _ |

**Table S3**. Effect of DMSO with 0.5% Tween on immobility of *N. aberrans* J2s after different exposure times

| Blank | 24h | 36h | 48h | 60h | 72h |
| --- | --- | --- | --- | --- | --- |
| 1 | 0.19±0.81 | 0.369±2 | -0.17±2.2 | 0.76±3.4 | 3.0±2.5 |
| 2 | 0.19±0.81 | 0.369±2 | -0.17±2.2 | 0.76±3.4 | 3.0±2.5 |
| 3 | 0.19±0.81 | 0.369±2 | -0.17±2.2 | 0.76±3.4 | 3.0±2.5 |
| 4 | 0.19±0.81 | 0.369±2 | -0.17±2.2 | 0.76±3.4 | 3.0±2.5 |
| 5 | 0.19±0.81 | 0.369±2 | -0.17±2.2 | 0.76±3.4 | 3.0±2.5 |
| 6 | 0.19±0.81 | 0.369±2 | -0.17±2.2 | 0.76±3.4 | 3.0±2.5 |

Each blank was replicated five times
